# Dynamics of phosphorus content and the activity of phosphatase in forest soil in the sustained nitrogen compounds emissions zone

**DOI:** 10.1007/s11356-018-3348-5

**Published:** 2018-10-02

**Authors:** Joanna Lemanowicz

**Affiliations:** Department of Biogeochemistry and Soil Science, Faculty of Agriculture and Biotechnology, UTP University of Science and Technology in Bydgoszcz, 6 Bernardyńska St., 85-029 Bydgoszcz, Poland

**Keywords:** Enzymes, Factors DI and AF, Forest, Nitrogen plant, Phosphorus, Soil

## Abstract

This paper summarizes research work on the seasonal and profile dynamics of phosphorus content and the activity of phosphatase in soil next to the nitrogen industry. The results are presented of the total phosphorus (TP) and available phosphorus (AP) content and the alkaline phosphatase (AlP) and acid phosphatase (AcP) against the basic physicochemical properties (clay, pH, total organic carbon, total nitrogen). Three soil profiles were sampled from Brunic Arenosols 0.8, 2.0, and 2.5 km away from the nitrogen plant. The control profile was taken from the Tuchola Forest. The soil was collected in both spring and autumn. The results showed that the total phosphorus content was higher in spring than in autumn (the value of index of changes in time TI < 0) contrary to available phosphorus (TI > 0) and in both seasons in surface soils, the lowest, in profile I. Both total and available phosphorus decreased with depth along the soil profiles. The distribution index (DI) calculated for total phosphorus in surface soils demonstrated a rather moderate accumulation, while DI value for available phosphorus for profile III, a considerable accumulation. The availability factor (AF) for all the soil samples was above the threshold of phosphorus load (2%) in the two seasons in this study (from 2.00 to 10.13% for spring and from 3.92 to 21.19% for autumn), suggesting that the transformation rate from TP to AP was high, and AP supply for plant growth was sufficient. The correlation analysis showed a significant and positive correlation of available phosphorus with soil properties such as total organic carbon (*r* = 0.577), total nitrogen (*r* = 0.512), and clay (*r* = 0.493); however, there was no correlation with the activity of phosphatases.

## Introduction

Phosphorus is an essential plant nutrient. Soils generally contain between 0.1 and 3.0 g P kg^−1^ soil. Total and available phosphorus are two important indicators to measure P levels in soil. The primary source of phosphorus in soils is minerals (especially apatites and hydroxylapatites) found in parent material. However, the remains of dead organisms, tree-crown water deposition, rainfall water deposition, and fertilizers constitute a secondary external and internal source of that element (Jonczyk et al. 2015). Phosphorus in forest ecosystems is a deficit nutrient and its content in soils depends on phosphorus abundance in plant litter. The availability of phosphorus occurring in organic bonds to plants depends on the rate of mineralization and not on the total content of those compounds. The study (Jonard et al. 2014) indicates that forest ecosystems lose their ability of efficient phosphorus recycling probably due to an excessive nitrogen input and climatic stress. The study by Wardle et al. (2004), Wassen et al. (2005), and Turner et al. (2013) shows that the total soil P gradually decreases as the result of weathering, and ecosystems may decline at their advanced stage, which results in a decrease in biomass and diversity due to soil P limitation. Many studies have reported that the dynamics of phosphorus in soil depend upon pH value (Kim et al. 2003), nitrogen or organic matter concentrations (Canellas et al. 2010), and soil clay content (Yu et al. 2016). According to Mosier and Zhu (2000), increasing available mineral nitrogen in soils leads to enhanced N_2_O formation and emission via increased nitrification and denitrification. In the research of Deng et al. (2017), nitrogen addition stimulated the sequestration of P in both plant and litter biomass. This may result in a significant decrease in soil phosphorus (Vitousek et al. 2010). The study on Zheng et al. (2017) showed that temperature is the most critical factor controlling the soil nitrogen, and species composition is the main factor regulating the soil available phosphorus.

The key role in the process of biochemical mineralization of organic phosphorus bonds is played by phosphatases (Eivazi and Tabatabai 1977; Nannipieri et al. 2011). The enzymes can be a good indicator of the potential of organic phosphorus mineralization as well as the biological activity of soil. In soil, biological (microbial and biochemical) activity plays a key role in nutrient cycling and amelioration in plant stresses and it is responsible for wide ecological functions of soil (Grover et al. 2011). Phosphatases, hydrolysing organic phosphorus compounds, are the most frequently investigated soil enzymes since they respond fastest to environmental stress caused by anthropogenic and natural factors. Unfortunately, a rapid development of industry globally is one of the main reasons of unfavorable changes in the right functioning of the ecosystem as well as soil environment (Telesiński et al. 2010). Until recently soil was regarded as an environmental filter ensuring the quality of both water and atmosphere (Trasar-Cepeda et al. 2000). According to Bálintová and Luptáková (2012), Shang et al. (2012), and Haddad et al. (2018), a full scale of the problem depends on the size of the polluted area, the depth at which pollutants penetrate soil, the chemical composition of polluting substances, and the different soil types.

The anthropogenic impact on the content of phosphorus in forest soils is hardly known. Little information was available on the vertical distribution of total and available phosphorous forms and the activity of phosphatase in the soil in the zone of sustained emissions of nitrogen compounds. Bearing that in mind, the hypothesis was made that long-term emissions of nitrogen compounds could less affect the phosphorus content and the activity of phosphatases in soil. The primary objective of this study was an analysis of the distribution of total and available phosphorus along the soil profiles in two sampling seasons and revealing the relationships between soil P and other selected soil properties.

## Material and methods

### Location of soil sampling

**Fig. 1:**
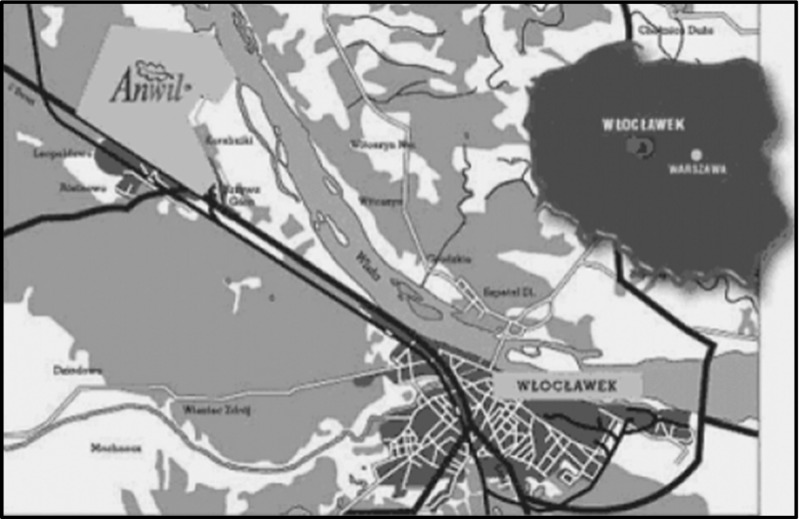
Localization of the study area

**Fig. 2:**
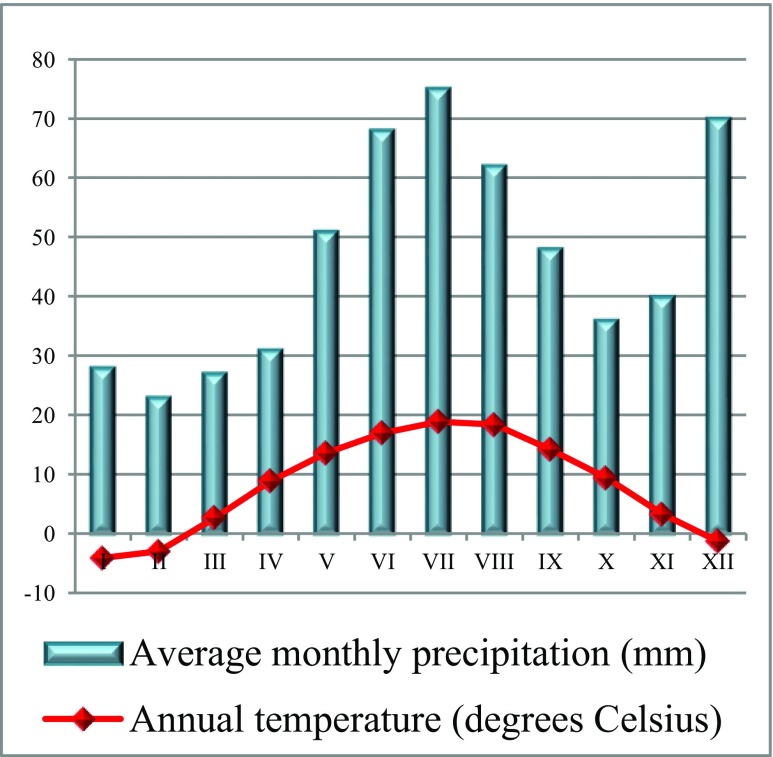
Annual temperature (°C) and average monthly precipitation (mm) for Włocławek

The research material was collected in spring and autumn (April and September) 2010, from 15 samples taken from four selected soil profiles representing one type of soil: *Brunic Arenosols* (IUSS WRB 2014). The study area is located in Włocławek (52° 41′ 55″ N, 18° 58′ 09″ E) (the Kujawy and Pomerania Province, central Poland) (Fig. 1). To carry out the study, soil samples were taken from the mineral horizons of three soil profiles from the sites adjacent to Anwil S.A. (the nitrogen plant) in its impacted area: profile I—approximately 0.8 km to the northwest; profile II—approximately 2 km west; profile III—approximately 2.5 km from the right bank of the Vistula River to the east. The profile of soil control located beyond the reach of the emissions was taken from a fresh mixed coniferous forest (BMśw) in Szumiąca, in the Tuchola Forest. The mean annual temperature in the study area is 8.6 °C, and the mean annual precipitation is 559 mm (Fig. 2). Anwil S.A. Nitrogen Plant in Włocławek, established in 1966, is one of Poland’s largest producers of nitrogen fertilizers (amongst others, ammonium nitrate and calcium ammonium nitrate). It also produces suspension polyvinyl chloride, chemical products for processing in a variety of industrial sectors, and agricultural products. The forests near the Plant are dominated by Scots pine (*Pinus sylvestris* L.), Silver birch (*Betula pendula* Roth.), and English oak (*Quercus robur* L.) growing in a fresh mixed coniferous forest habitat (BMśw) and in the lowest undergrowth—*Vaccinium myrtillus*, *Convallaria majalis*, and *Entodon schreberi*.

### Soil analysis

Each soil sample was air-dried at room temperature until receiving a solid mass, crushed, and sieved to separate the fraction < 2 mm from gravel or larger detritus. The following soil properties and components were determined: total organic carbon (TOC), using the Scalar Company’s TOCN FORMACTSTM analyzer; total nitrogen (TN) with the Kjeldahl method (ISO 11261, 2002); pH in H_2_O and in 1 M solution of KCl (ISO 10390:1997); total phosphorus (TP) with the method by Mehta et al. (1954), by treating soil with concentrated HCl; and then with 0.5 M NaOH. After mixing, the extracts were mineralized with a respective mixture of concentrated acids: HNO_3_ acid (V), HClO_4_ (VII), and H_2_SO_4_ (VI) at the ratio of 10:1:4. After mineralization, the optical density was assayed at 660 nm using Spectrophotometer Marcel Pro. The content of available phosphorus (AP) was determined with the Egner-Riehm method-DL (Egner et al. 1960), which involves the spectrophotometric measurement of the intensity of the color of phosphorus-molybdenum blue, produced by orthophosphoric ions with molybdenum ions in the acid environment, in the presence of SnCl_2_. The soil alkaline phosphatase (AlP) [E.C. 3.1.3.1] and acid phosphatase (AcP) [E.C. 3.1.3.2] activities were measured based on the detection of p-nitrophenol (PNP) released after incubation (37 °C, 1 h) at pH 6.5 for acid phosphatase and pH 11.0 for alkaline with p-nitrophenyl phosphate disodium (Tabatabai and Bremner 1969).

Based on the results, the index of changes in time was calculated:

1 $$ TI=\frac{t2}{t1} $$

where *t*1 is the content of the element in spring; *t*2 is the content of the element in autumn. TI > 1 means an increase, and TI < 1 means a decrease in the content of carbon and phosphorus and the activity of phosphatase (Lemanowicz and Krzyżaniak 2015).

The availability factor (AF) for phosphorus, as suggested by Obrador et al. (2007), was applied for this purpose. It is expressed as follows:

2 $$ \mathrm{AF}=\left(\frac{\mathrm{AP}}{\mathrm{TP}}\right)\times 100 $$

The distribution of the elements in the soil profiles was described with the value of the distribution index (DI), calculated adequately (Kobierski and Dąbkowska-Naskręt 2012),

3 $$ \mathrm{DI}=\frac{\mathrm{the}\ \mathrm{content}\ \mathrm{of}\ \mathrm{the}\ \mathrm{element}\ \mathrm{in}\ \mathrm{the}\ \mathrm{solum}\ \mathrm{horizon}}{\mathrm{the}\ \mathrm{content}\ \mathrm{of}\ \mathrm{the}\ \mathrm{element}\ \mathrm{in}\ \mathrm{the}\ \mathrm{parent}\ \mathrm{material}} $$

where DI < 1 stands for a lack of anthropogenic impact, 1 ≤ DI ≤ 3 stands for a moderate accumulation, 3 ≤ DI ≤ 6 stands for a considerable accumulation, and DI > 6 stands for a very high accumulation.

### Statistical analysis

The paper presents the arithmetic means of the results. Besides, the results of the analyses were exposed to the analysis of simple correlation (*P* < 0.05) that determined the degree of dependence between respective features. The analysis of the correlation was made using “Statistica for Windows Pl” software (Poland). In this study, some physical and chemical properties in soil were analyzed applying the multivariate analyses*.* The principal component analysis (PCA) was used to identify the properties which account for most of the variability and to select the most appropriate indicators that influence the soil quality.

## Results

### Physical and chemical properties

Basic granulometric analysis is presented in Table 1. The dominant fraction in the soil samples studied was the sand fraction from 0.05 to 2.0 mm in diameter (84–98%). The content of clay fraction (particle size < 0.002 mm) ranged from 1 to 9%. The samples were classified only as two grain-size groups: loose and slightly loamy sands (according to USDA).

**Table 1:** Physical and chemical properties in soil

Depth (cm)	Horizon	Clay (%)	pH		TOC (g kg^−1^)		TN (g kg^−1^)		TOC/TN	
			H_2_O	KCl	Spring	Autumn	Spring	Autumn	Spring	Autumn
Control										
0–20	A	9	5.71	5.11	12.09	22.36	0.871	1.431	13.9	15.6
20–50	ABv	5	5.71	5.13	4.16	5.616	0.312	0.390	13.4	14.4
50–100	BvC	5	5.09	4.25	0.754	0.702	0.071	0.052	10.8	14.0
100–130	C	1	5.10	4.31	0.624	0.507	0.065	0.042	10.4	12.7
Profile I										
0–15	A	1	5.34	4.46	11.44	10.61	0.981	0.821	11.7	12.9
15–72	Bv	1	5.59	4.99	1.040	1.989	0.913	0.203	1.10	9.90
72–100	C	1	5.61	4.09	0.416	0.440	0.042	0.065	10.4	7.40
Profile II										
0–15	A	3	5.43	4.72	8.879	19.66	0.922	1.511	9.7	13.0
15–39	ABv	1	5.66	4.98	4.888	5.252	0.530	0.515	9.2	10.3
39–87	C1	4	5.73	4.98	0.507	0.455	0.064	0.052	8.5	9.10
87–100	C2	3	5.85	4.97	0.195	0.338	0.021	0.044	9.8	8.50
Profile III										
0–4	A	7	5.35	4.39	5.850	24.99	0.390	1.510	15.0	16.6
4–18	ABv	1	5.60	4.93	1.976	7.878	0.144	0.670	14.1	11.8
18–60	Bv	2	6.04	5.17	1.963	2.756	0.153	0.234	13.1	12.0
60–150	C	1	6.61	5.41	0.650	0.468	0.061	0.051	10.8	9.40

*TOC* total organic carbon, *TN* total nitrogen

Marking the exchangeable and hydrolytic acidity indicates acidic and very acidic soil. The values expressed in H_2_O pH ranged from 5.09 to 5.71, while in 1 mol KCl, from 4.25 to 5.11 (profile control) and from 5.34 to 6.61 (pH H_2_O in profiles I–III) and from 4.39 to 5.41 (pH KCl in profiles I–III) (Table 1).

The content of total organic carbon in all the profiles regularly decreases with depth, falling within the range 0.624–12.09 g kg^−1^ and 0.507–22.36 g kg^−1^ (in the profile control, respectively in spring and autumn) and 0.195–11.44 and 0.339–24.99 g kg^−1^ (in the profiles within the impact of the nitrogen plant, respectively in spring and autumn) (Table 1). The same regularities in the vertical distribution were identified for nitrogen (Table 1). The calculated values of DI for TOC and TN point to a clear tendency to a very high accumulation in the surface horizons of the soil profiles (> 6), which is related to anthropogenic effect (Table 3). An inconsiderable range of the values of the ratio TOC/TN (8.5–16.6) shows a high biological activity of the soils, irrespective of the distance from the emitter (Table 1). The value of the ratio TOC/TN in the range 10–17 stands for poorly degraded soil. A vast majority of organic matter penetrating forest soils undergoes the process of mineralization.

### The content of phosphorus

The total phosphorus content in the soils was similar, falling within the range from 0.320 to 0.376 g kg^−1^ (spring) and from 0.157 to 0.239 g kg^−1^ (autumn) in the control profile and from 0.220 to 0.437 g kg^−1^ (spring) and from 0.123 to 0.240 g kg^−1^ (autumn) in soil affected by the nitrogen plant (Table 2). In this study, the profile control woodland showed a higher TP content than the profiles affected by the nitrogen plant (Table 2). With the total phosphorus content in spring and in autumn, the index of time (TI) was below 0 (TI < 0) (Table 2), which points to the total phosphorus content being lower in autumn. Similar results are reported by Gao et al. (2016). The value of DI for TP (in horizon A) indicates a moderate accumulation (Table 3).

**Table 2:** The content of total phosphorus (TP) and available phosphorus (AP), the availability factor (AF), and index of time (TI)

Depth (cm)	Horizon	TP (g kg^−1^)		AP (mg kg^−1^)		AF (%)		TI	
		Spring	Autumn	Spring	Autumn	Spring	Autumn	TP	AP
Control									
0–20	A	0.376	0.239	24.45	39.10	6.50	16.36	0.65	1.60
20–50	ABv	0.331	0.180	14.58	38.11	4.41	21.17	0.54	2.61
50–100	BvC	0.320	0.301	12.24	28.65	3.82	9.52	0.94	2.34
100–130	C	0.324	0.157	9.33	22.54	2.88	12.35	0.48	2.42
Profile I									
0–15	A	0.250	0.208	12.74	17.00	5.09	8.17	0.83	1.33
15–72	Bv	0.344	0.202	15.38	18.97	4.47	9.39	0.59	1.23
72–100	C	0.301	0.123	6.01	8.50	2.00	6.91	0.41	1.42
Profile II									
0–15	A	0.255	0.231	20.18	26.16	7.91	11.32	0.91	1.30
15–39	ABv	0.437	0.194	23.27	29.75	5.32	15.33	0.44	1.28
39–87	C1	0.286	0.179	18.27	23.89	6.31	13.34	0.63	1.32
87–100	C2	0.261	0.138	12.73	16.21	4.48	11.74	0.53	1.27
Profile III									
0–4	A	0.248	0.216	25.13	35.64	10.13	16.50	0.87	1.42
4–18	ABv	0.331	0.240	30.35	34.19	9.20	14.24	0.73	1.12
18–60	Bv	0.306	0.189	11.68	17.92	3.81	9.47	0.62	1.53
60–150	C	0.220	0.191	5.29	7.49	2.40	3.92	0.87	1.42

**Table 3:** Distribution index value (DI)

Depth (cm)	Horizon	TOC		TN		TP		AP	
		Spring	Autumn	Spring	Autumn	Spring	Autumn	Spring	Autumn
Control									
0–20	A	19.38	44.10	14.5	35.8	1.16	1.522	2.621	1.735
20–50	ABv	6.67	11.08	5.2	9.8	1.02	1.146	1.563	1.691
50–100	BvC	1.21	1.38	1.2	1.3	0.988	1.917	1.312	1.271
Profile I									
0–15	A	27.50	24.00	24.5	37.8	0.831	1.691	2.120	2.000
15–72	Bv	2.50	4.50	22.8	3.3	1.143	1.642	2.559	2.232
Profile II									
0–15	A	45.53	58.15	46.0	37.8	0.977	1.674	1.585	1.614
15–39	ABv	25.07	15.54	3.8	12.8	1.674	1.406	1.828	1.835
39–87	C1	2.60	1.35	3.0	1.3	1.096	1.297	1.425	1.474
Profile III									
0–4	A	9.00	53.42	6.5	30.2	1.127	1.131	4.750	4.758
4–18	ABv	3.04	16.83	2.3	13.4	1.505	1.257	5.737	4.565
18–60	Bv	3.02	5.89	2.5	4.6	1.391	0.990	2.208	2.393

*TOC* total organic carbon, *TN* total nitrogen, *TP* total phosphorus, *AP* available phosphorus

The content of available phosphorus in soils varied depending on the distance from the nitrogen source (Table 2). The lowest AP content (from 6.01 to 15.38 mg kg^−1^) was found in the soil profile located closest to the nitrogen plant (profile I—0.8 km). The content of available phosphorus was increasing with the distance from the emitter and it was highest in profile III (from 5.29 to 25.13 mg kg^−1^) in the control profile soil, which classifies the soil as IV and V class with a low and very low content of that element (PN-R-04023: 1996). A positive correlation between the soil AP content and TN (*r* = 0.512; *P* = 0.262) was recorded. A higher AP content in all the soil profiles was observed in autumn at each of the four sampling sites than in spring. It is confirmed by the results of TI the value of which in all the profiles was above 1 (TI > 1) (Table 2). It can be related to less phosphorus uptake by plants and plant litter breakdown as well as to a return to surface soils in autumn. The values of DI for AP in horizon A of profiles I–II and the control ranged from 1.585 to 2.621, which points to a moderate accumulation. The highest DI values were reported in profile III (4.750 in spring and 4.758 in autumn), which indicates its considerable accumulation (Table 3).

Based on the TP and AP in soil, the AF, also known as the coefficient of phosphorus activation, was calculated (Table 2). The AF ratios for all the soil samples were above the threshold of P load (2%) for P bioavailability (Xiao et al. 2012) in the two seasons in this study (from 2.00 to 10.13% for spring and from 3.92 to 21.19% for autumn), suggesting that the transformation rate from TP to AP was high, and AP supply for plant growth was enough. The AF exhibited similar profile distributions to AP in two seasons.

### Alkaline and acid phosphatase

The highest activity of alkaline phosphatase was reported in soil profile I and the control, and the activity of acid phosphatase, in profile I (Table 4), which was due to soil reaction. The activity of phosphatases in the soil sampled from the control point corresponded to the level of activity in the soil of profile III. The activity of phosphomonoesterases was decreasing with the depth of soil profiles. The tendency is related to a spatial distribution of humus and soil microorganisms and a decreasing amount of carbon substrates available both to microorganisms and to enzymes, which coincides with the TOC values in soil (Table 1).

**Table 4:** The activity of alkaline phosphatase (AlP) and acid phosphatase (AcP) and index of time (TI)

Depth (cm)	Horizon	AlP		AcP		TI	
		(mM pNP kg^−1^ h^−1^)					
		Spring	Autumn	Spring	Autumn	AlP	AcP
Control							
0–20	A	2.933	0.942	5.578	2.207	0.32	0.40
20–50	ABv	0.870	0.474	1.373	0.776	0.55	0.57
50–100	BvC	0.472	0.319	1.891	1.682	0.68	0.89
100–130	C	0.267	0.198	0.474	0.410	0.74	0.86
Profile I							
0–15	A	2.171	2.033	9.906	7.217	0.92	0.73
15–72	Bv	1.050	0.607	2.595	1.682	0.58	0.65
72–100	C	0.575	0.165	0.791	0.489	0.29	0.62
Profile II							
0–15	A	1.690	0.744	7.174	4.335	0.44	0.60
15–39	ABv	0.921	0.821	5.082	3.055	0.89	0.60
39–87	C1	0.997	0.409	1.157	0.877	0.41	0.76
87–100	C2	0.574	0.305	0.812	0.352	0.53	0.43
Profile III							
0–4	A	2.746	2.092	6.506	4.155	0.76	0.64
4–18	ABv	1.229	0.776	1.768	1.121	0.63	0.63
18–60	Bv	0.439	0.266	0.748	0.295	0.61	0.39
60–150	C	0.208	0.295	0.704	0.582	1.41	0.83

The phosphatase activity showed the highest value in spring and the lowest in autumn, seen from the values of the index of time (TI < 0) (Table 4). The linear regression equation shows that with an increase in the content of total organic carbon in soil by 1.0 g, there was an activation of alkaline phosphatase which increased by 0.0621 mM pNP kg^−1^ h^−1^ and acid phosphatase, by 0.201 mM pNP kg^−1^ h^−1^. With the calculated coefficient of determination (*R*^2^) it was found that only 30.5% of variation in the activity of AlP and 28.90% is accounted for by the variation in TOC, whereas the other about 70%, by other soil parameters (Table 5). The activity of phosphatases was decreasing with the depth of soil profiles. The phosphatase activity was inhibited by a lack of nutrients due to the total organic carbon in the topsoil layer being higher than that in the subsoil layer. The soil acid phosphatase activity demonstrated a significantly positive correlation with total N (*r* = 0.627; *P* = 0.0002). Additional nitrogen increases the plant and microbial productivity and thus increases a demand for phosphorus. The research showed that alkaline and acid phosphatase were all significantly positively correlated with each other (*r* = 0.834; *P* = 0.00001), indicating that any enzyme activity can reflect other enzyme activities in soil essentially. In our study, no significant relationship between alkaline and acid phosphatase activity and any of phosphorus forms was observed.

**Table 5:** Pearson’s correlation coefficients (*n* = 30)

Variables		Equation	*r*	*R* ^2^	*P*
Dependent	Independent				
Total organic carbon	Total nitrogen	*y* = − 0.5343 + 13.426*x*	0.933	0.871	0.00001
Available phosphorus	Total organic carbon	*y* = 15.87 + 0.811*x*	0.577	0.333	0.0008
Acid phosphatase	Total organic carbon	*y* = 1.457 + 0.201*x*	0.538	0.289	0.0021
Alkaline phosphatase	Total organic carbon	*y* = 0.588 + 0.0621*x*	0.552	0.305	0.0015
Total organic carbon	Clay	*y* = 2.007 + 0.186*x*	0.511	0.262	0.0039
Available phosphorus	Total nitrogen	*y* = 15.684 + 10.337*x*	0.512	0.262	0.0038
Alkaline phosphatase	Total nitrogen	*y* = 0.514 + 0.928*x*	0.574	0.329	0.0009
Acid phosphatase	Total nitrogen	*y* = 1.062 + 3.362	0.627	0.362	0.0002
Alkaline phosphatase	Clay	*y* = 0.502 + 0.139*x*	0.451	0.202	0.0124
Available phosphorus	Clay	*y* = 14.486 + 1.90*x*	0.493	0.243	0.0055
Acid phosphatase	Alkaline phosphatase	*y* = −0.0115 + 2.763*x*	0.834	0.693	0.00001

**Fig. 3:**
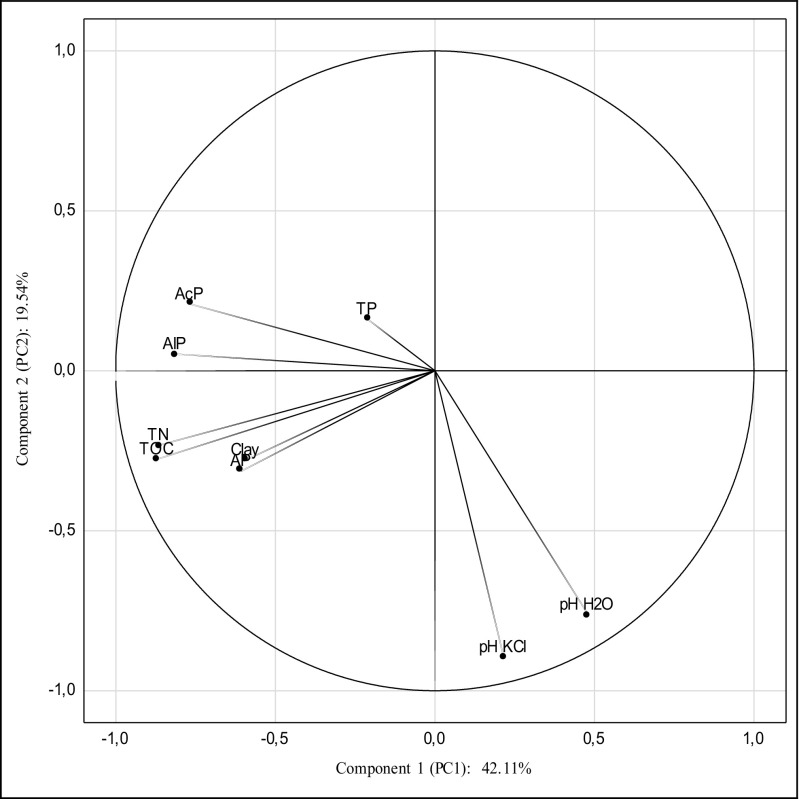
Configuration of variables in the system of the two axes of principal components PC1 and PC2 of the content of total organic carbon (TOC), total nitrogen (TN), total (TP) and available (AP) phosphorus, alkaline (AlP), and acid (AcP) phosphatase activities and physicochemical properties in soil

To determine the nature and the strength of the bonds between soil properties, the content of the macroelements, and the activity of phosphatases, the PCA was applied. Table 6 indicates factor loadings as well as the Eigen values. Two principal components (PC1 and PC2) were extracted from the available dataset that explained a total variance of approximately 61.64% (Fig. 3). Component 1 (PC1) is responsible for 42.11% of the total element variables and indicated a great negative correlation with total organic carbon (− 0.865), total nitrogen (− 0.864), alkaline (− 0.813), and acid (− 0.767) phosphatase. These four elements may reflect the anthropogenic contamination of soil. As for the activity of alkaline and acid phosphatase, it was found that most variances contained in the first principal component (PC1) were positively correlated with the content of TOC and TN, and negatively with the soil reaction. Component 2 (PC2) accounts for 19.53% and it is dominated only by pH in H_2_O (− 0.762) and in KCl (− 0.894).

**Table 6:** Values of the three extracted factor loadings for nine elements

Elements	PC1	PC2
pH H_2_O	0.480	− 0.762*
pH KCl	0.215	− 0.894*
Total organic carbon	− 0.865*	− 0.275
Total nitrogen	− 0.864*	− 0.232
Total phosphorus	− 0.211	− 0.161
Available phosphorus	− 0.605	− 0.312
Alkaline phosphatase	− 0.813*	0.052
Acid phosphatase	− 0.767*	0.208
Clay	− 0.591	− 0.278
Variation (%)	42.11	19.53

*Statistically significant

## Discussion

The acid soils are common in forest ecosystems and pH influences the transformation of organic matter in soil (Tonon et al. 2010). The acidification of soils in the vicinity of the nitrogen plant was due to many years of emissions of nitrogen oxides, sulphur dioxide, and ammonium in a form of wet and dry deposition. Increased nitrogen emissions to forest soils result in a release of protons H^+^. They are not balanced during plant material mineralization, which contributes to secondary effects of soil acidification.

The content of total phosphorus in soils depends considerably on the primary richness of parent materials and a modifying impact of a soil factors complex (Jonczyk et al. 2015). According to Tripathi et al. (2008), atmospheric phosphorus deposition might elevate TP levels in soil. According to Liu et al. (2014), nitrogen could provide the necessary element for the production of phosphatase catalysing biochemical phosphorus mineralization. In this study, the soil total N content accounted for about 60% of the variations in soil available phosphorus. In soil profile I, nitrogen exacerbated soil acidification. This decreases soil P availability by binding phosphate ions with Al and Fe (Tian and Niu 2015; Devau et al. 2009). The content of available phosphorus generally decreased along the soil profiles in two sampling seasons, which was in agreement with the study of Ye et al. (2014) and Gao et al. (2016). Li et al. (2017) showed that temperature, atmospheric precipitation, and vegetation may well explain the variation of the content of soil carbon, nitrogen, and phosphorus. According to Li and Zhang (1994), it is considered an essential soil fertility factor. Some studies demonstrated that higher AF in soils could promote plant growth (Zhao et al. 2009; Xiao et al. 2012). The study of Zheng et al. (2017) showed that the pine-dominated forests had the highest content of phosphorus in soil, while the mixed forests and oak-dominated forests had slightly lower content P. This was confirmed by this study.

According to many authors (Bielińska et al. 2013; Bartkowiak et al. 2017; Błońska et al. 2017; Onyszko et al. 2017; Lemanowicz et al. 2018), the enzymatic tests facilitate the evaluation of both the effect of natural factors and the human impact on ecosystem functioning. The highest activity of acid phosphatase resulted from the fact that phosphomonoesterases are enzymes most susceptible to changes in soil reaction (Dick et al. 2000), the optimum pH of soil for the activity of alkaline phosphatase was 9.0–11.0, and for acid phosphatase, 4.0–6.5. Similar research results are presented by Kotroczó et al. (2014). The seasonal changes in the alkaline and acid activity of phosphatase are related to both the changes in the availability of nutrients in soil and hydrothermal conditions. In the study by Kang and Freeman (1999), the phosphatase activity correlated closely with soil temperature, soil water content, and pH, and spring is the time when soil temperature and moisture might be optimal. According to Olander and Vitousek (2000), a high content of N may stimulate the activity of phosphatases since nitrogen is essential for some enzyme synthesis. In the study reported by Margalef et al. (2017), it was found that TN was the most important factor affecting the activity of phosphatase. Studies by Deng et al. (2017) showed enhanced phosphatase activity (+ 24%) by the addition of nitrogen. This suggests that nitrogen content is likely to promote mobilization of phosphorus, which is supported by the increase in phosphatase activity. However, the activity of those enzymes was also associated with climatic and soil type. The interaction of many different parameters suggests that phosphorus cycling is driven by a broad-scale pattern of the ecosystem productivity capacity. Similar results were reported by Orczewska et al. (2012). Some other factors may influence the enzyme activity, for example, the content of organic matter, nitrogen availability, or the interaction between enzyme and clay, and heavy metals. According to Zheng et al. (2018), the enzyme activities differ significantly across different types of forests. The soil of mixed forests identifies a higher total and available nitrogen, total phosphorus, available potassium, and total organic carbon contents. Mixed forests show a 95.9% higher total abundance of soil microorganisms and 104.5% higher counts of bacteria than pure-stand forests (Yu et al. 2015).

## Conclusion

The seasonal and vertical dynamics of soil total and available phosphorus were investigated in the zone of sustained emissions of nitrogen compounds.

- It was shown that the parameters of forest soils differed depending on the distance from the nitrogen plant. There was found an increase in the activity of alkaline activity with the increase in the distance from the nitrogen plant, which was accompanied by favorable changes in the content of carbon of organic compounds and available phosphorus.
- The values of TOC/TN, TI, AF, DI presented a direction of phosphorus transformations depending on anthropogenic and hydrothermal factors.
- The content of available phosphorus in the soil was low and very low (based on the classes of richness in that element).The results showed that the available phosphorus content was higher in autumn than in spring (the value of index of changes in time TI > 0).
- The value of DI for horizon A of profiles I–II points to a moderate accumulation, while the accumulation of available phosphorus in profile III was considerable.
- The AF (the availability factor) for all the soils was above the threshold of P load (2%) for P bioavailability in two seasons in this study, suggesting that the transformation rate from TP to AP was high, and the AP supply for plant growth was sufficient.

Dynamic transformations which occur in forest ecosystems as a result of changing environment conditions require a continuous enhancement and further development of knowledge on the transformations of phosphorus compounds in soil. Knowing the degree of soil phosphorus mineralization by evaluating the activity of phosphomonoesterases would allow for increasing the content of mineral phosphorus in soil.
